# Soil mixture composition alters Arabidopsis susceptibility to *Pseudomonas syringae* infection

**DOI:** 10.1002/pld3.44

**Published:** 2018-02-19

**Authors:** Jana A. Hassan, Roberto de la Torre‐Roche, Jason C. White, Jennifer D. Lewis

**Affiliations:** ^1^ Department of Plant and Microbial Biology University of California Berkeley Berkeley CA USA; ^2^ Department of Analytical Chemistry The Connecticut Agricultural Experiment Station New Haven CT USA; ^3^ Plant Gene Expression Center United States Department of Agriculture Albany CA USA

**Keywords:** *Arabidopsis thaliana*, environment, host resistance, *Pseudomonas syringae*, salicylic acid, soil

## Abstract

*Pseudomonas syringae* is a gram‐negative bacterial pathogen that causes disease on more than 100 different plant species, including the model plant *Arabidopsis thaliana*. Dissection of the *Arabidopsis thaliana*–*Pseudomonas syringae* pathosystem has identified many factors that contribute to successful infection or immunity, including the genetics of the host, the genetics of the pathogen, and the environment. Environmental factors that contribute to a successful interaction can include temperature, light, and the circadian clock, as well as the soil environment. As silicon‐amended Resilience soil is advertised to enhance plant health, we sought to examine the extent to which this soil might affect the behavior of the *A. thaliana*–*P. syringae* model pathosystem and to characterize the mechanisms through which these effects may occur. We found that plants grown in Si‐amended Resilience soil displayed enhanced resistance to bacteria compared to plants grown in non‐Si‐amended Sunshine soil, and salicylic acid biosynthesis and signaling were not required for resistance. Although silicon has been shown to contribute to broad‐spectrum resistance, our data indicate that silicon is not the direct cause of enhanced resistance and that the Si‐amended Resilience soil has additional properties that modulate plant resistance. Our work demonstrates the importance of environmental factors, such as soil in modulating interactions between the plant and foliar pathogens, and highlights the significance of careful annotation of the environmental conditions under which plant–pathogen interactions are studied.

## 1 INTRODUCTION

*Pseudomonas syringae*, a gram‐negative, rod‐shaped bacterium, infects a wide range of species, including the model plant, *Arabidopsis thaliana*. Under cool moist conditions, *P. syringae* causes substantial disease and is spread to adjacent plants or leaves by rain splash (Hirano & Upper, 2000). Disease depends on compatibility among the host, pathogen, and environment, in an interaction termed the disease triangle (Agrios, 2005). Host genetics determine whether the plant is resistant or susceptible to the pathogen, and pathogen genetics determine whether the pathogen is virulent or nonvirulent to a specific host (Dangl, Horvath, & Staskawicz, 2013; Dangl & Jones, 2001; Vleeshouwers & Oliver, 2014). Environmental factors play a substantial role in contributing to the outcome of a plant–pathogen interaction, and these conditions can vary depending on the plant and pathogen under study.

Plants protect themselves from pathogens with three layers of induced defenses. In the first line of defense, the plant utilizes transmembrane pattern recognition receptors (PRRs) to recognize highly conserved pathogen‐associated molecular patterns (PAMPs), such as bacterial flagellin (Zipfel et al., 2004). PRR‐triggered immunity (PTI) results in the activation of mitogen‐activated protein kinase cascades, production of reactive oxygen species, induction of pathogenesis‐related genes, and deposition of callose and stomatal closure (Macho & Zipfel, 2014). To suppress PTI, pathogens such as *Pseudomonas syringae* can use the type III secretion system (T3SS) to mobilize type III‐secreted effector proteins (T3SEs) across the cell wall and into the host cell (Gohre & Robatzek, 2008; Grant, Fisher, Chang, Mole, & Dangl, 2006; Lewis, Desveaux, & Guttman, 2009; Mudgett, 2005; Xin & He, 2013). Common targets of T3SEs are PRRs, mitogen‐activated protein kinase (MAPK) cascades associated with PTI, vesicular trafficking pathways involved in the transport of antimicrobial compounds, and hormone signaling pathways (Block & Alfano, 2011; Feng & Zhou, 2012; Grant et al., 2006; Lewis et al., 2009; Mudgett, 2005; Shigenaga & Argueso, 2016; Speth, Lee, & He, 2007; Zhou & Chai, 2008). In the second line of defense, the plant uses nucleotide‐binding leucine‐rich repeat receptors (NLRs, also called Resistance proteins) which can recognize specific effectors resulting in effector‐triggered immunity (ETI) (Jones, Vance, & Dangl, 2016; Schreiber, Baudin, Hassan, & Lewis, 2016). ETI often culminates in a hypersensitive response (HR), characterized by localized programmed cell death at the infection site (Heath, 2000). In the third line of defense, plants are primed to respond quickly to subsequent pathogen exposure, resulting in systemic acquired resistance (SAR) (Fu & Dong, 2013; Gao, Kachroo, & Kachroo, 2014; Gao, Zhu, Kachroo, & Kachroo, 2015). SAR allows plants to more effectively fend off a subsequent pathogen attack and induces the production of salicylic acid (SA), a hormone which plays a central role in immune responses. While SA is a major contributor to systemic broad‐spectrum resistance in plants, complex interactions with other plant hormones also contribute to immunity (Shigenaga & Argueso, 2016).

Environmental factors such as light, temperature, humidity, water availability, and soil both separately and in combination profoundly affect the molecular mechanisms underlying plant defense responses to pathogens (Bostock, Pye, & Roubtsova, 2014; Hua, 2013; Smirnova et al., 2001; Suzuki, Rivero, Shulaev, Blumwald, & Mittler, 2014). Light is necessary for immunity to viral, bacterial, and fungal pathogens, including the development of the HR and SA‐mediated defense responses (Chandra‐Shekara et al., 2006; Griebel & Zeier, 2008; Lozano & Sequeira, 1970; Roden & Ingle, 2009). Ambient temperature influences PTI and ETI responses. Plants favor PTI responses at higher temperatures (23–32°C) which inhibit bacterial effector secretion and promote bacterial proliferation (Cheng et al., 2013; van Dijk et al., 1999). Plants activate ETI signaling at lower temperatures (10–23°C) when bacteria secrete effectors to promote virulence (Cheng et al., 2013; van Dijk et al., 1999). Both light and temperature are important external cues that entrain the plant circadian clock, which also plays an integral role in coordinating immune responses to pathogens. For both bacterial and fungal pathogens, plants are more susceptible when infected at subjective morning as opposed to at night (Bhardwaj, Meier, Petersen, Ingle, & Roden, 2011; Lu, McClung, & Zhang, 2017; Wang et al., 2011). Mutations in core clock genes abolish temporal variation in resistance levels, suggesting that clock genes play an important role in plant immunity (Bhardwaj et al., 2011; Lu et al., 2017; Wang et al., 2011). Lastly, abiotic and biotic soil factors affect plant resistance to disease. Abiotic factors such as water availability or nutrient availability in the soil can impact plant immunity (Bostock et al., 2014; De Coninck, Timmermans, Vos, Cammue, & Kazan, 2015; Suzuki et al., 2014; Thalineau et al., 2016). The soil microbiome can provide the host with protection from disease (Bakker, Doornbos, Zamioudis, Berendsen, & Pieterse, 2013; Pieterse et al., 2014), by competing with the pathogen for host nutrients, interacting antagonistically with the pathogen (antibiosis) or eliciting induced systemic resistance (ISR).

We identified enhanced resistance to *P. syringae* in Arabidopsis grown on commercial soil enriched with silicon. Using the Arabidopsis–*P. syringae* pathosystem, we investigated the effects of silicon‐amended soil (Resilience) compared to non‐silicon‐amended soil (Sunshine) and characterized the mechanisms that underlie these effects. We show that Si‐amended Resilience soil enhances plant resistance to multiple strains of the foliar pathogen, *P. syringae*, when it is inoculated into the apoplast and to a moderately virulent *P. syringae* strain that is spray‐inoculated onto the leaf surface. Plants impaired in PTI still exhibited enhanced resistance, and ETI responses are normal in plants grown in amended Resilience soil. In addition, the observed resistance is mediated by SA‐independent pathways. Interestingly, this work demonstrates that the soil environment can influence plant resistance to a foliar pathogen and points to the importance of carefully documented conditions for plant–pathogen interactions.

## 2 MATERIALS AND METHODS

### 2.1 Bacterial strains and routine culture conditions

*Pseudomonas syringae* was grown in either King's broth (KB) or minimal medium for induction of the type III secretion system (Huynh, Dahlbeck, & Staskawicz, 1989). Antibiotics were used at the following concentrations: 50 μg/ml kanamycin, 50 μg/ml rifampicin (*Pto*DC3000, *Pma*M6C∆E, and *Pto*DC3000∆*hrcC*), 300 μg/ml streptomycin (*Pcal*ES4326), and 50 μg/ml cycloheximide. *Pto*DC3000 carrying HopZ1a under its native promoter with a C‐terminal in‐frame HA epitope tag was previously described (Lewis, Abada, Ma, Guttman, & Desveaux, 2008).

### 2.2 Plant growth conditions

*Arabidopsis thaliana* plants were grown under 9 hrs of light (~130 microeinsteins m^−2^ s^−1^) and 15 hrs of darkness at 22°C in Sunshine #1 (Sun) or Sunshine Resilience #1 (Res) soil (Sun Gro Horticulture) supplemented with 20:20:20 fertilizer. Sunshine #1 soil is the previous formulation of Resilience and is not amended with silicon. Sunshine #1 soil must be ordered as a custom formulation. Resilience soil is reported to be amended with 1.7 mM silicon (Sun Gro Horticulture); however, its contents are patented. Assays used ecotype Col‐0 as the wild‐type background, the following mutants: *npr1‐1* (Cao, Bowling, Gordon, & Dong, 1994), *ics1* (Dewdney et al., 2000; Nawrath & Metraux, 1999; Wildermuth, Dewdney, Wu, & Ausubel, 2001), or the transgenic line *nahG* (Delaney et al., 1994). Plants were grown for 5–6 weeks before evaluating them for silicon accumulation, HR, or in bacterial growth assays; 5–6 pools of three individuals were used for silicon accumulation experiments. A total of 8–10 individuals were used for bacterial growth assays; 17–30 individuals were used for biomass accumulation experiments.

### 2.3 *P. syringae* HR and in planta growth assays

*Pseudomonas syringae* was resuspended in 10 mM MgCl_2_ to an optical density at 600 nm of 0.1 (~5 × 10^7^ cfu/ml) for HR assays, or an optical density at 600 nm of 0.8 (~4.0 × 10^8 ^cfu/ml) for spray growth assays, or diluted to a concentration of 1 × 10^5^ cfu/ml for growth assays by pressure infiltration. For HR and growth assays, bacteria were hand‐infiltrated into the leaf using a needleless syringe as described previously (Katagiri, Thilmony, & He, 2002). For HR, the plants were infiltrated in the late afternoon and maintained under 24 hrs of light. The HR was scored at 16–20 hrs. For infiltrated growth assays, four disks (total of 1 cm^2^) were harvested, ground in 10 mM MgCl_2_, and plated on KB with rifampicin or streptomycin and cycloheximide on days 0, 3, or 7 (*Pma*M6C∆E) to count colonies. For spray assays, the bacterial inoculum included 0.02% Silwet L‐77, and the plants were sprayed until the leaf surface was completely wet. The plants were covered with a humidified dome for 3 or 7 (*Pma*M6C∆E) days. On day 3 or 7 (*Pma*M6C∆E), the leaves were sterilized in 70% ethanol for 10 s and rinsed in sterile H_2_O for 10 s before harvesting, grinding, and plating as above. For infiltrated growth assays or spray assays, the plants were inoculated in the morning and kept in the growth chamber under short‐day conditions during the infections.

### 2.4 *Pseudomonas syringae* protein expression

*Pseudomonas syringae* cultures were grown overnight in KB containing kanamycin and rifampicin, pelleted, and washed in minimal media. Bacteria were resuspended in minimal media supplemented with potassium silicate (adjusted to pH 5.7 with H_3_PO_4_) at a final concentration of 1 μM, 10 μM, 100 μM, 500 μM, or 1 mM. Cultures were incubated with shaking at 28°C overnight to induce the type III secretion system (Huynh et al., 1989). An aliquot of 1.3 ml of each culture was pelleted, resuspended in 50 μl of 1× Laemmli loading dye, and boiled for 5 min, and 5 μl was separated on 12% sodium dodecyl sulfate‐polyacrylamide (SDS‐PAGE) gels. Proteins were transferred to nitrocellulose membranes and detected with HA antibodies (Roche) by chemiluminescence (GE Healthcare).

### 2.5 Silicon analysis

Sequential digestion steps (acid and alkaline) were conducted in vessels for a CEM Mars 6 Microwave Digestor (Buckingham, UK). Oven‐dried plant samples (approximately 100 mg) were digested by microwave‐assisted digestion (set at 1,300 W) using 5 ml of 1 M HNO_3_ plus 5 ml of H_2_O_2_ (30% v/v) (Barros, de Souza, Schiavo, & Nobrega, 2016). The heating program was as follows: 5 min to reach 120°C with a hold for 5 min, 5 min to reach 160°C with a hold for 5 min, and 3 min to reach 210°C for 5 min. The vessels were then removed and cooled to ambient temperature. Each vessel was then amended with 5 ml of 1.5 M NaOH and heated as follows: 10 min to reach 150°C with a 5 min hold; 10 min to reach 200°C with a 10 min hold. After cooling, the vessels were amended with 750 μl of 14 M HNO_3_. Final volumes were adjusted to 50 ml prior to analysis by inductively coupled plasma mass spectrometry (ICP‐MS; Agilent 7500ce) for Si. The digest Si content was quantified against a four‐point calibration curve that had been previously evaluated for linearity and accuracy. Analytical blanks, matrix blanks, and calibration verification samples were included in each sequence.

### 2.6 Soil analyses

Analyses were performed by the Cornell Nutrient Analysis Laboratory using standard methods.

### 2.7 Statistical analyses

The data were analyzed using two‐tailed homoscedastic *t* tests in Minitab 17. A significance level of α = 0.05 was chosen for all statistical analyses.

## 3 RESULTS

### 3.1 Resistance to virulent *P. syringae* strains in the apoplast is enhanced in plants grown in silicon‐amended Resilience soil

**Figure 1:**
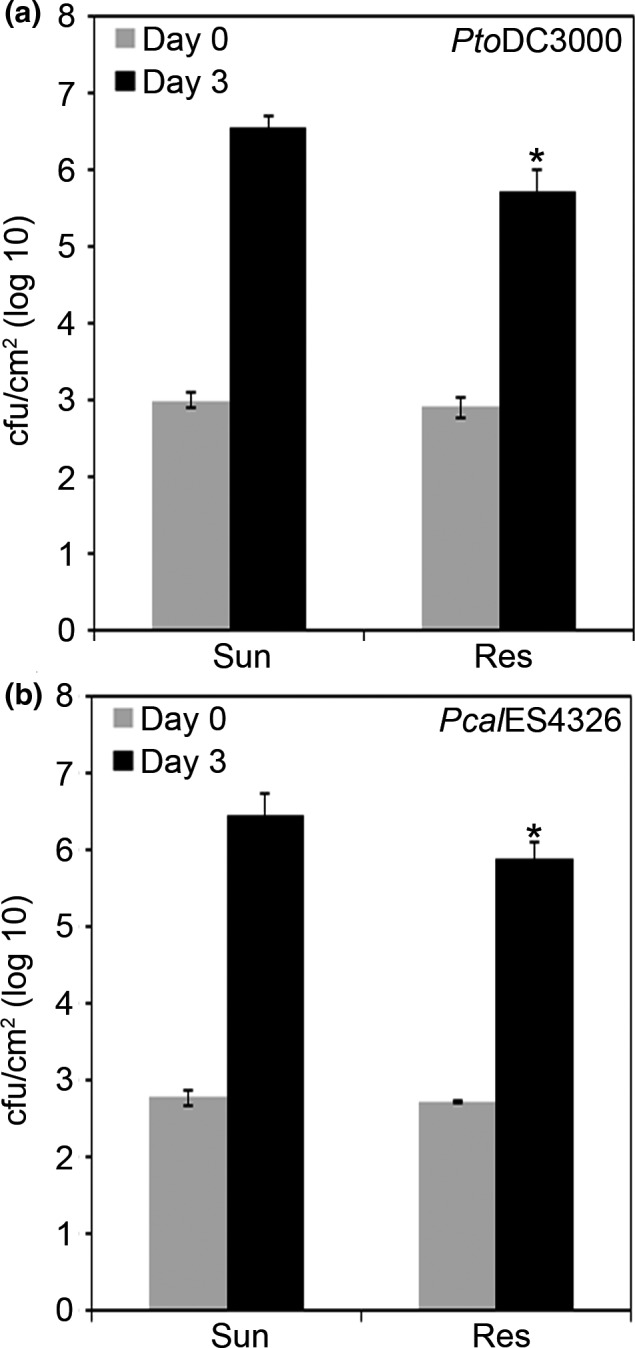
*Pseudomonas syringae* strains exhibit reduced virulence after pressure infiltration into plants grown on Resilience soil. (a) *P. syringae* pv. *tomato* DC3000 (*Pto* DC3000) was syringe infiltrated into Arabidopsis ecotype Col‐0 leaves, with a suspension containing 1 × 10^5^ CFU/ml. Bacterial counts were determined 1 hr postinfection (day 0) and 3 days postinfection (day 3). (b) *P. cannabina* pv. *alisalensis* ES4326 (*Pcal* ES4326) was syringe infiltrated as described above. Error bars indicate the standard deviation. Two‐tailed homoscedastic t tests were performed to test for significant differences. Significant differences are shown with an asterisk (*p < .001). Experiments were repeated three times with similar results

To investigate whether Arabidopsis plants grown in silicon‐amended Resilience soil or non‐silicon‐amended Sunshine soil show differential growth of virulent *P. syringae* strains, we carried out bacterial growth assays with two different strains, *P. syringae pv. tomato* DC3000 (*Pto*DC3000) or *P. cannabina* pv. *alisalensis* ES4326 (*Pcal*ES4326, formerly *P. syringae pv. maculicola* ES4326 *Pma*ES4326). Bacteria were pressure‐infiltrated into the leaves of 5‐week‐old Arabidopsis plants that were grown in Sunshine (non‐silicon‐amended) or Sunshine soil amended with silicon (hereafter Resilience), and the bacterial populations were monitored at 1 hr (day 0) and 3 days after infiltration (day 3). In plants grown on Sunshine soil, both strains grew to high levels (~log 6–7), indicating that the pathogens were able to cause disease (Figure 1a, b). In plants grown in Resilience soil, both strains exhibited statistically significant reductions in growth (0.5–1 log) (Figure 1a, b).

**Figure 2:**
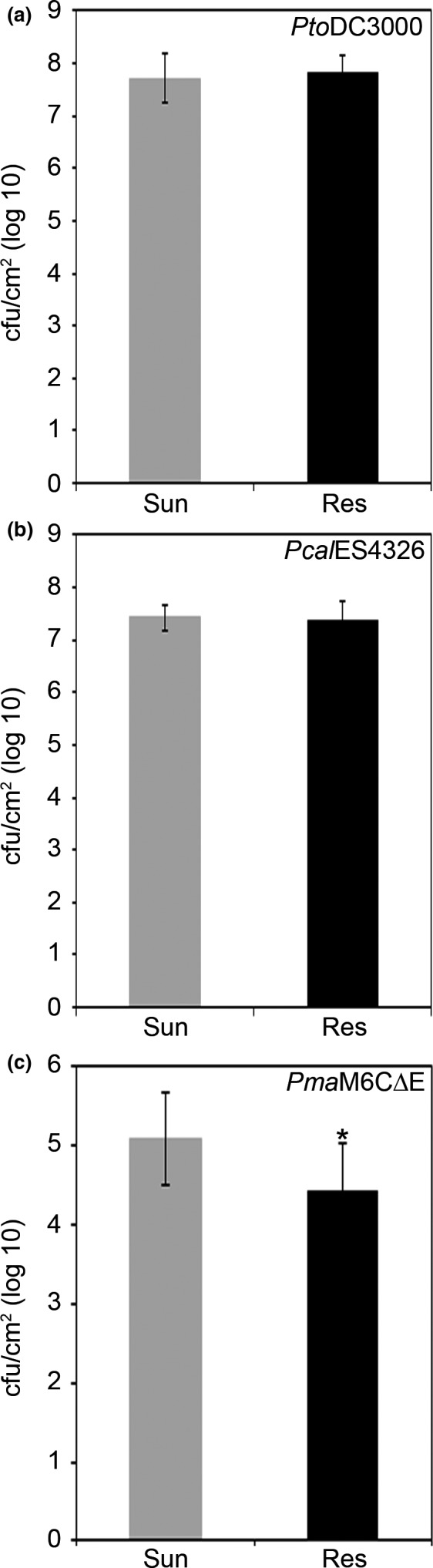
Highly virulent *P. syringae* strains show similar virulence in plants grown in Sunshine or Resilience soil after spray infiltration, while a moderately virulent *P. syringae* strain exhibits restricted bacterial growth on plants grown in Resilience soil. Strains were spray‐inoculated onto Arabidopsis ecotype Col‐0 leaves, with a suspension containing ~4.0 × 10^8^ CFU/ml and 0.02% Silwet. Error bars indicate the standard deviation. Two‐tailed homoscedastic t tests were performed to test for significant differences. (a) Bacterial counts for *P. syringae* pv. *tomato* DC3000 (*Pto* DC3000) were determined 3 days postinfection. No significant differences were observed. Experiments were repeated three times with similar results. (b) Bacterial counts for *P. cannabina* pv. *alisalensis* ES4326 (*Pcal* ES4326) were determined 3 days postinfection. No significant differences were observed. Experiments were repeated three times with similar results. (c) Bacterial counts for *P. syringae* pv. *maculicola* M6C∆E (*Pma*M6C∆E) were determined 7 days postinfection. Significant differences are shown with an asterisk (*p < .05). Experiments were repeated two times with similar results

To test whether plants grown in Resilience or Sunshine soil exhibited differential entry of bacteria into the leaf, we also infected Arabidopsis plants by spray inoculation, which requires the bacteria to swim into the leaf through the stomata. Bacteria were sprayed onto the leaves of 5‐week‐old Arabidopsis plants, and the endophytic bacterial populations were monitored 3 days after inoculation. We observed high levels of bacterial growth (~log 7–8) with *Pto*DC3000 (Figure 2a) or *Pcal*ES4326 (Figure 2b), in plants grown on Sunshine or Resilience soil. To determine whether the high bacterial titers associated with *Pto*DC3000 and *Pcal*ES4326 may have overcome the subtle resistance benefit conferred by growth of the plants in Resilience soil, we also carried out spray assays with the moderately virulent *P. syringae pv. maculicola* M6C∆E (hereafter *Pma*M6C∆E) strain (Rohmer, Kjemtrup, Marchesini, & Dangl, 2003). As *Pma*M6C∆E is not highly infectious when sprayed onto the leaves, we maintained the plants in high humidity conditions and monitored the endophytic bacterial populations 7 days after inoculation. We observed a significant reduction of 0.5–1 log bacterial growth in plants grown with on Resilience soil compared to plants grown on Sunshine soil (Figure 2c). Therefore, plants grown in Resilience soil showed resistance against bacteria inoculated into the apoplast or onto the surface of the leaf, indicating that bacterial entry was not affected by the plants' soil environment. The observed resistance was dependent on the bacterial titer and could be overcome by highly virulent strains.

### 3.2 Pattern‐triggered immunity (PTI) does not contribute to enhanced resistance in plants grown in Resilience soil

**Figure 3:**
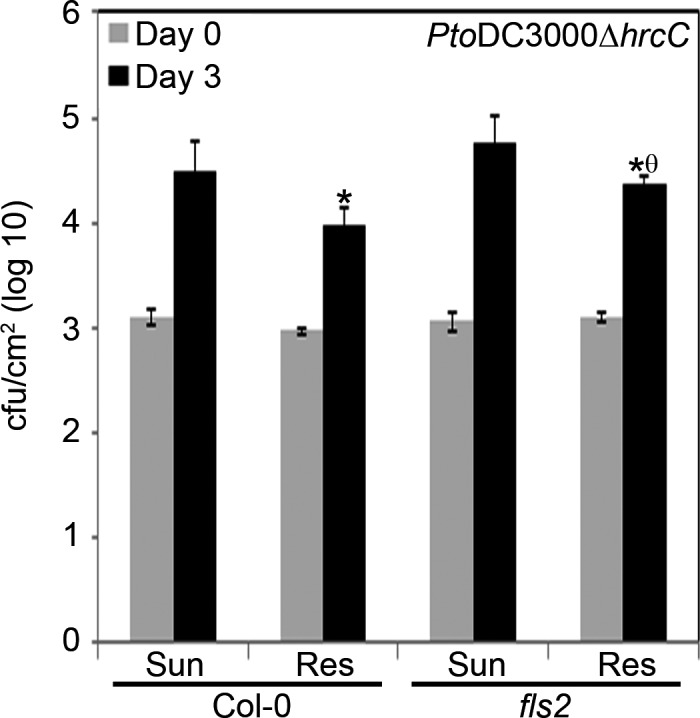
PTI does not contribute to the enhanced resistance observed in plants grown in Resilience soil. *P. syringae* pv. *tomato* DC3000∆*hrcC* (*Pto* DC3000∆*hrcC*) was syringe infiltrated into Arabidopsis ecotype Col‐0 or *fls2* leaves, with a suspension containing 1 × 10^5^ CFU/ml. Bacterial counts were determined 1 hr postinfection (day 0) and 3 days postinfection (day 3). Error bars indicate the standard deviation. Two‐tailed homoscedastic t tests were performed to test for significant differences. Significant differences between genotypes on the same soil type are shown with an asterisk (*p < .05), and significant differences between soil types for the same genotype are shown with a theta (θ *p* < .05). The experiment was repeated two times with similar results

To determine whether plants grown in Resilience soil are affected in PRR‐triggered immune responses, we carried out bacterial growth assays with a strain of *P. syringae pv. tomato* DC3000*∆hrcC* (hereafter *Pto*DC3000*∆hrcC*) that is unable to secrete type III effector proteins, due to a mutation in a structural component of the type III secretion system (Roine et al., 1997). We tested wild‐type Col‐0 and the *fls2* mutant which lacks the PRR necessary for recognition of bacterial flagellin (Gomez‐Gomez & Boller, 2000; Gomez‐Gomez, Felix, & Boller, 1999). We found that both Col‐0 and *fls2* plants grown in Resilience soil supported ~0.4–0.5 log less bacterial growth than plants grown in Sunshine soil (Figure 3), indicating that PTI does not contribute to the restriction of bacterial growth. As expected, *Pto*DC3000*∆hrcC* growth was slightly greater in *fls2* compared to Col‐0 on both soil types (Figure 3), as the flagellin peptide is not recognized. These data indicate that the plants grown in Resilience soil have enhanced resistance that is not PTI‐dependent.

### 3.3 Plants grown in Resilience soil exhibit typical effector‐triggered immunity (ETI)

**Figure 4:**
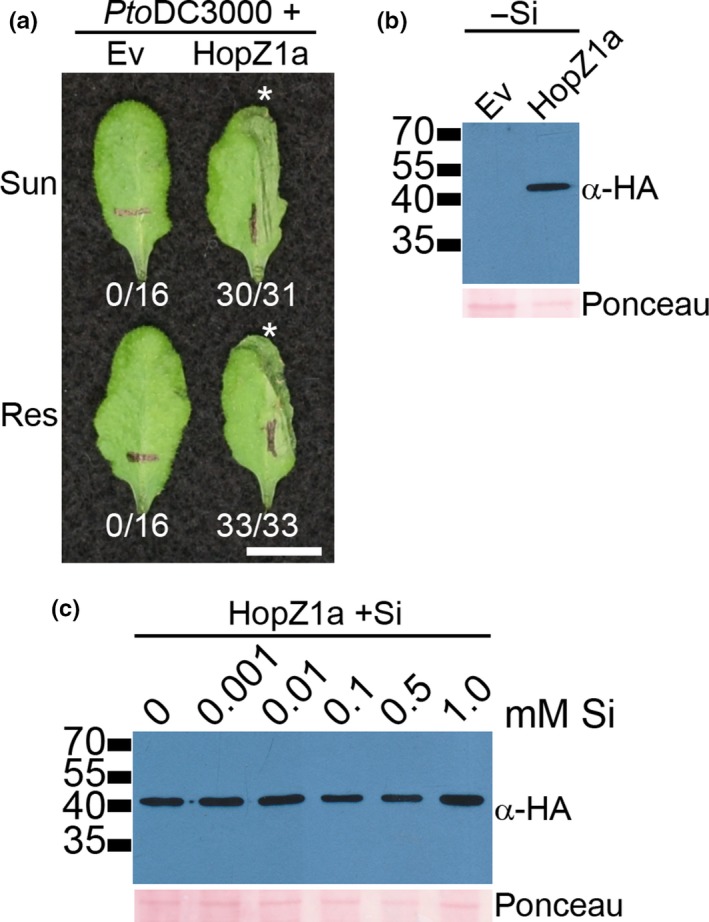
Plants grown in Resilience soil show normal ETI responses, and *P. syringae* effector production is not affected by silicon. (a) Half‐leaves of Arabidopsis ecotype Col‐0 were infiltrated with 5 × 10^7^ CFU/ml of *P. syringae* pv. *tomato* DC3000 expressing empty vector (*Pto* DC3000 (*Ev*)) or HopZ1a with a C‐terminal HA tag under the control of its endogenous promoter (*Pto* DC3000 (*hopZ1a*)). Photographs were taken 22 hrs after infiltration. The HR is indicated with an asterisk. The number of leaves showing an HR is shown below each treatment. Scale bar = 1 cm. (b) Immunoblot analysis of HopZ1a‐HA protein expression in *P. syringae* pv. *tomato* DC3000 (*Pto* DC3000). *Pto* DC3000 carrying an empty vector (Ev) or HA‐tagged HopZ1a (42.1 kDa) was grown in minimal media to induce the type III secretion system. Equal amounts of proteins were separated on 12% SDS‐PAGE gels, blotted onto nitrocellulose, and probed with HA antibodies. The Ponceau red‐stained blot was used as the loading control. (c) Immunoblot analysis of HopZ1a‐HA protein expression in *P. syringae* pv. *tomato* DC3000 (*Pto* DC3000) as in part b. *Pto* DC3000 carrying HA‐tagged HopZ1a was grown in minimal media, with different concentrations of potassium silicate pH 5.7 as indicated

We also investigated whether plants grown in Resilience soil are impacted in ETI by conducting hypersensitive response (HR) assays with HopZ1a, a well‐characterized bacterial effector that causes a strong immune response in Arabidopsis ecotype Col‐0 (Lewis, Wu, Guttman, & Desveaux, 2010; Lewis et al., 2008). The HR is visualized as silvering and flattening of the infiltrated half‐leaf within 16–20 hrs postinfiltration (Lewis et al., 2008, 2010). Plants grown in Sunshine or Resilience soil showed similarly strong HRs in the same time frame (16–20 hrs postinfiltration) (Figure 4a), suggesting that the soil in which plants are grown does not affect ETI.

### 3.4 *P. syringae* effector HopZ1a is induced regardless of silicon treatment

Vivancos, Labbe, Menzies, and Belanger (2015) recently suggested that silicon might affect effector secretion or function in the apoplast. As Resilience soil is amended with silicon, we investigated whether silicon could interfere with effector expression. As the amount of effector proteins produced during *P. syringae* infection *in planta* is exceedingly low, we tested whether silicon could impact effector expression in vitro using minimal medium that induces the type III secretion system (Huynh et al., 1989; Lewis et al., 2008) and minimal medium containing a range of different concentrations of potassium silicate at pH 5.7. The pH of the minimal medium and the carbon source are particularly important for effector production (Huynh et al., 1989). We examined protein expression of T3SE HopZ1a tagged with a hemagglutinin (HA) tag, as we have previously demonstrated that it, like other T3SEs, is secreted and translocated into plant cells (Lewis et al., 2008). HopZ1a was expressed in *P. syringae* grown in standard minimal media lacking potassium silicate and visualized by Western blot analysis (Figure 4b; Lewis et al., 2008). We tested whether HopZ1a production was affected when *P. syringae* was grown in minimal medium containing 1 μM to 1 mM potassium silicate, as the maximum solubility of silicon is 1.7 mM. Similar levels of HopZ1a protein were detected across the range of potassium silicate concentrations (Figure 4c). This suggests that silicon does not impair effector production in vitro.

### 3.5 Plants grown in Resilience soil do not accumulate more silicon

**Figure 5:**
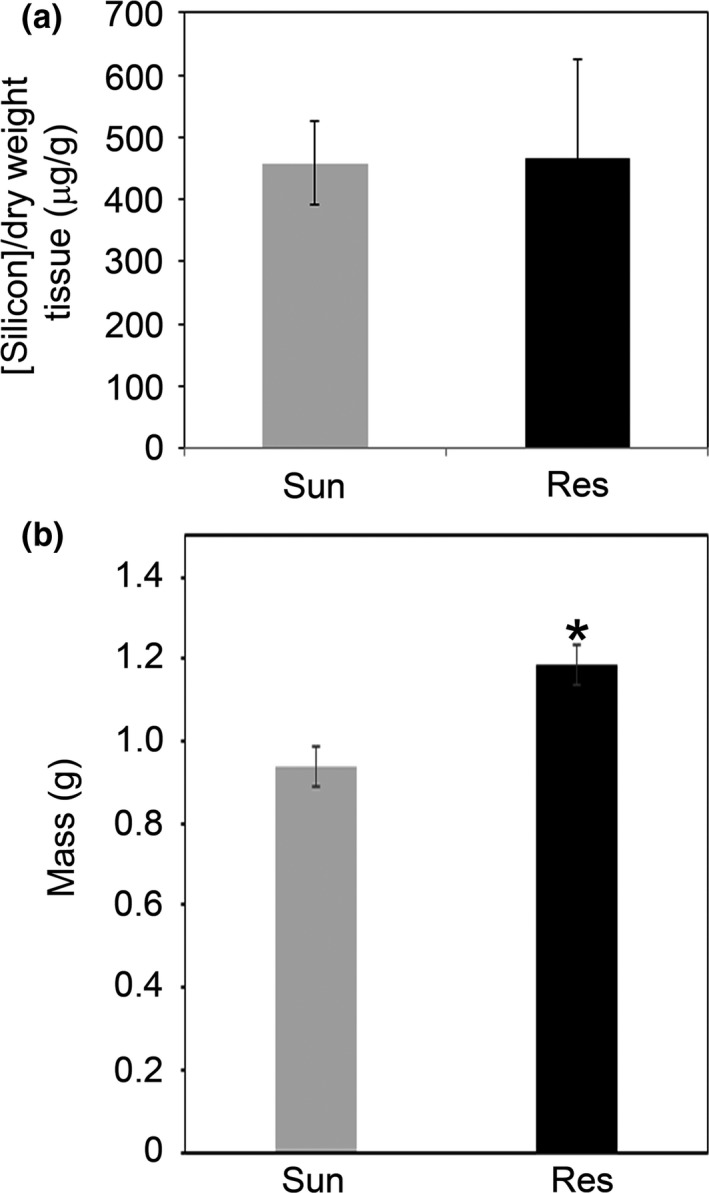
Plants grown in Resilience soil do not accumulate more silicon but do accumulate more biomass. Arabidopsis ecotype Col‐0 plants were initially grown on 0.5× Murashige and Skoog plates for 10 days, before being transplanted to Sunshine or Resilience soil. (a) The average level of silicon accumulation after 5–6 weeks is shown. Error bars indicate the standard error from the mean of six individuals. Two‐tailed homoscedastic t tests were performed to test for significant differences. No significant differences were observed. The experiment was repeated two times with similar results. (b) The average aerial biomass for each plant, 5–6 weeks after transplanting, is shown. Error bars indicate the standard error from the mean of 26 individuals. Two‐tailed homoscedastic t tests were performed to test for significant differences. Significant differences are shown with an asterisk (*p < .05). The experiment was repeated three times with similar results

Resilience soil is reported to be Sunshine soil that is amended with 1.7 mM silicon, but is patented so its contents are not readily ascertained. Silicon has been extensively characterized for its beneficial effects on disease resistance to biotrophic and necrotrophic pathogens, as well as on abiotic stress (Datnoff, Deren, & Snyder, 1997; Epstein, 1994, 1999; Guerriero, Hausman, & Legay, 2016; Liang, Nikolic, Belanger, Gong, & Song, 2015a, 2015b; Van Bockhaven et al., 2015). However, Arabidopsis has been reported to accumulate low levels of silicon and to lack silicon influx transporters (Ghanmi, McNally, Benhamou, Menzies, & Belanger, 2004). To determine whether there was differential uptake of silicon in plants grown in Sunshine or Resilience soil, we conducted inductively coupled plasma mass spectrometry (ICP‐MS), which allows for the quantification of trace elements (Kroukamp, Wondimu, & Forbes, 2016). Plants grown in Sunshine versus Resilience soil accumulated similar levels of silicon (Figure 5a), although the range of silicon concentrations was much broader in the plants grown in Resilience soil. These data suggest that silicon is not responsible for the enhanced resistance to *P. syringae* and that additional unknown factors in Resilience soil contribute to plant resistance to *P. syringae* infection.

We observed that Arabidopsis plants were frequently bigger when grown on Resilience soil compared to Sunshine soil. To determine whether Arabidopsis would exhibit enhanced biomass accumulation on Resilience soil, we grew Arabidopsis seedlings on Resilience or Sunshine soil for 5–6 weeks under short‐day conditions. To avoid any effects of soil type on germination, we sowed out Arabidopsis seed on Murashige and Skoog media, incubated the plates at 4°C to synchronize seed germination, and transplanted 10‐day‐old seedlings to Resilience or Sunshine soil. The average aerial biomass was significantly different between the two treatments. Plants grown on Sunshine soil had an average biomass of 0.94 g, while plants grown on Resilience soil had an average biomass of 1.18 g, representing a 25% increase in biomass (Figure 5b).

To identify other potential differences between Sunshine and Resilience soils, we carried out comprehensive soil analysis. Sunshine and Resilience soils contained similar amounts of total carbon, total nitrogen, and cation exchange capacity, which measures the soil's capacity to bind cations (Table 1). Both contained a high percentage of organic matter, and Resilience soil contained more organic matter (~74%) than Sunshine soil (~65%) (Table 1) using the loss on ignition method (Nelson & Sommers, 1996). Sunshine soil was slightly less acidic (pH 6.02) than Resilience soil (pH 5.25). Sunshine soil contained less moisture (7.99%) than Resilience soil (12.16%) (Table 1). We also measured macronutrients, micronutrients, and heavy metals in the two soil types, for bioavailable elements and for total element content (Table 2). Resilience soil contained more total calcium (Res 32,363 mg/kg vs. Sun 19,166 mg/kg), and more total sodium (Res 1349 mg/kg vs. Sun 794 mg/kg), compared to Sunshine soil; however, the bioavailable calcium and sodium contents were similar. Resilience soil contained more total potassium (Res 2,185 mg/kg vs. Sun 1,494 mg/kg), more total magnesium (Res 13,471 mg/kg vs. Sun 2,766 mg/kg), and more total sulfur (Res 5,273 mg/kg vs. Sun 4,547 mg/kg); however, bioavailable potassium, magnesium, and sulfur were higher in Sunshine soil (K 1,427 mg/kg, Mg 4,034 mg/kg, and S 2,643 mg/kg) versus Resilience soil (K 1,118 mg/kg, Mg 1,751 mg/kg, S 2,135 mg/kg). Bioavailable silicon was also higher in Resilience soil (45.01 mg/kg) compared to Sunshine soil (37.16 mg/kg). The observed differences were not particularly striking; however, our data suggest the differences in the soil itself contribute to the enhanced resistance.

**Table 1:** Properties of Sunshine and Resilience soil

	Moisture (%)	pH	Cation exchange capacity^a^	Organic matter (LOI, %)	Organic matter (%)	Total *N* (%)	Total C (%)
Sunshine	7.99	6.02	27.24	64.91	45.20	0.88	34.45
Resilience	12.16	5.25	30.88	74.05	51.60	0.93	37.69

LOI, loss on ignition method.

a Cation exchange capacity (CEC) measures the soil's capacity to hold cations, particularly potassium, calcium, magnesium, and sodium.

**Table 2:** Elemental analysis of Sunshine and Resilience soil

mg/kg	Al	As	B	Ba	Be	Ca	Cd	Co	Cr	Cu	Fe	K	Li	Mg
Bioavailable elements^a^														
Sunshine	10.09	0.74	1.88	5.80	–	11,337	0.05	0.10	0.25	0.23	6.17	1,427	–	4,034
Resilience	12.05	0.75	1.48	6.07	–	12,004	0.06	0.11	0.19	0.12	6.39	1,118	–	1,751
Total elements in soil^b^														
Sunshine	1,123	1	12.1	38	0	19,166	0	0.7	1	12	1240	1,494	5.9	2,766
Resilience	575	0	15.2	27	0	32,263	0	0.4	1	13	926	2,185	5.6	13,471

mg/kg	Mn	Mo	Na	Ni	P	Pb	S	Se	Si	Sr	Ti	V	Zn	
Bioavailable elements^a^														
Sunshine	27.31	0.16	359	0.30	446	0.38	2,643	–	37.16	28.96	–	–	5.55	
Resilience	32.09	0.17	286	0.29	512	0.32	2,135	–	45.01	30.58	–	–	4.56	
Total elements in soil^b^														
Sunshine	112.8	2.7	794	1	668	1	4,547	0	–	141.6	12.5	0.6	31	
Resilience	150.4	2.1	1349	0	660	1	5,273	0	–	142.0	9.2	0	29	

a Elements are available for uptake by plants.

b Total elements in soil include soluble and insoluble portions.

### 3.6 Plants grown on Resilience soil are not primed for salicylic acid‐dependent immune responses

**Figure 6:**
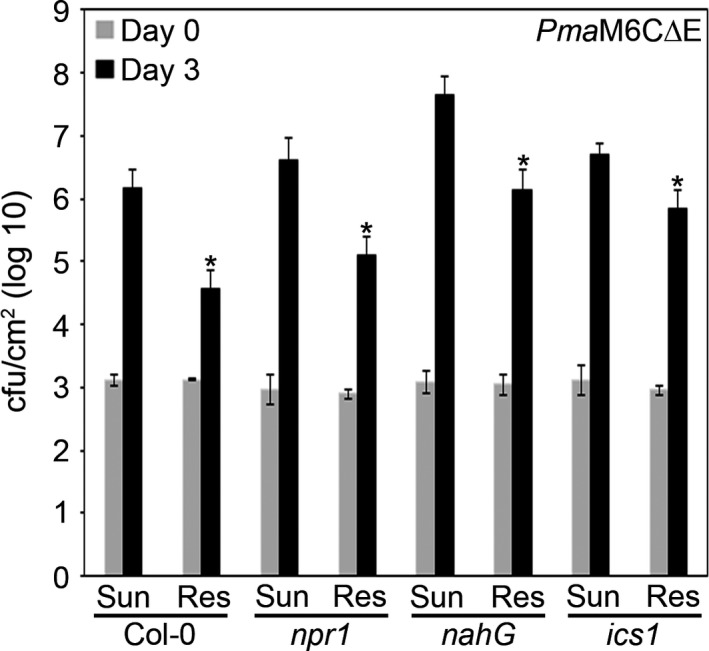
Plants grown in Resilience soil are not primed for salicylic acid‐dependent immune responses. *P. syringae* pv. *maculicola* M6C∆E (*Pma*M6C∆E) was syringe infiltrated into Arabidopsis ecotype Col‐0 leaves, *nahG*,* npr1,* or *ics1* mutants, with a suspension containing 1 × 10^5^ CFU/ml. Bacterial counts were determined 1 hr postinfection (day 0) and 3 days postinfection (day 3). Error bars indicate the standard deviation. Two‐tailed homoscedastic t tests were performed to test for significant differences in bacterial growth within a genotype on the two soil types. Significant differences are shown with an asterisk (*p < .001). The experiment was repeated two times with similar results

To investigate whether SA might contribute to priming of defenses in plants grown in Sunshine or Resilience soil, we conducted bacterial growth assays on Arabidopsis lines *(npr1, nahG*, or *ics1/sid2*) that are affected in SA production or signaling. As all three lines support higher bacterial growth than Col‐0 (Cao et al., 1994; Delaney et al., 1994; Lewis et al., 2010; Nawrath & Metraux, 1999), we used the moderately virulent *Pma*M6C∆E strain so that we could quantify impaired or enhanced virulence (Rohmer et al., 2003) and pressure‐infiltrated the bacteria. NPR1 is a master regulator of SA‐dependent defenses (Cao et al., 1994; Fu & Dong, 2013). *nahG* is a transgenic Arabidopsis line carrying the bacterial salicylate hydroxylase enzyme and cannot accumulate SA (Delaney et al., 1994). ICS1/SID2 is required for synthesis of SA in the chloroplast (Dewdney et al., 2000; Nawrath & Metraux, 1999; Wildermuth et al., 2001). In Col‐0, we observed ~log 6 growth in plants grown in Sunshine soil but only ~log 4.5 growth in plants grown in Resilience soil (Figure 6). As previously reported, *npr1*,* nahG*, and *ics1/sid2* lines were more susceptible to bacterial infections (Cao et al., 1994; Delaney et al., 1994; Lewis et al., 2010; Nawrath & Metraux, 1999). In *npr1*,* nahG*, and *ics1/sid2* lines, we observed ~log 6.5–7.5 growth in plants grown in Sunshine soil compared to ~log 5–6 growth in plants grown in Resilience soil (Figure 6). As the *npr1*,* nahG*, and *ics1/sid2* lines grown in Resilience soil still exhibited ~0.5–1.5 log lower bacterial growth compared to the same lines grown in Sunshine soil, this indicates that these SA‐related genes are not required for the restriction in bacterial growth. These results suggest that SA signaling does not contribute to enhanced resistance in plants grown in Resilience soil.

## 4 DISCUSSION

Although environmental effects on host resistance to pathogens have been well‐characterized, there has been little systematic evaluation of the contribution of soil to resistance and defense signaling in a well‐characterized pathosystem. We found that Arabidopsis plants grown in a silicon‐amended soil (Resilience) exhibit enhanced resistance toward the foliar pathogen *P. syringae* (Figure 1) and investigated how resistance was influenced by the type of soil in which the plants were grown. We observed enhanced resistance when *Pto*DC3000 or *Pcal*ES4326, two highly virulent strains of *P. syringae*, was inoculated into the apoplast of Arabidopsis plants grown in Resilience soil compared to Sunshine soil (Figure 1). We also tested plants grown in Resilience or Sunshine soil by spray inoculation, which requires that the bacteria swim into the leaf to establish infection. We found that resistance to *P. syringae* in plants grown on Resilience soil could be overcome by high bacterial titers (i.e., *Pto*DC3000 or *Pma*ES4326, Figure 2), while moderately virulent strains such as *Pma*M6C∆E (Gimenez‐Ibanez et al., 2014; Rohmer et al., 2003) exhibited restricted growth (reduction of ~0.5–1 log) in plants grown in Resilience soil (Figure 2).

Pathogen‐associated molecular patterns such as flg22 and Ef‐Tu are very common targets for the plant immune system (Zipfel et al., 2004, 2006). PAMPs are typically recognized by receptor‐like kinase (RLK) PRRs (Monaghan & Zipfel, 2012; Zipfel et al., 2004, 2006). RLKs are part of multigene families that function in many aspects of plant development and physiology and act as PRRs (Shiu et al., 2004). Only a handful of PAMPs and their cognate PRRs have been identified. We employed a type III secretion system mutant (*Pto*DC3000∆*hrcC*) that is unable to secrete effector proteins into its host, to test whether PTI is impacted by the soil that the plants were grown in. We tested Arabidopsis Col‐0 and the *fls2* mutant, which lacks the FLS2 PRR required for flg22 recognition (Gomez‐Gomez & Boller, 2000; Gomez‐Gomez et al., 1999). We found that the virulence of *Pto*DC3000∆*hrcC* was reduced by ~0.5 log in Arabidopsis Col‐0 or *fls2* grown in Resilience soil compared to plants grown in Sunshine soil (Figure 3), indicating that PTI does not contribute to the enhanced resistance. As expected, *Pto*DC3000∆*hrcC* also grew to higher bacterial titers in *fls2* compared to Col‐0 in both soil types, although these data were only statistically significant in Resilience soil (Figure 3). These data indicate that the resistance observed in plants grown in Resilience soil does not depend on PTI.

Silicon has been hypothesized to impair the function or perhaps secretion of effector proteins in the apoplast (Vivancos et al., 2015). To test these hypotheses, we infiltrated Arabidopsis with *Pto*DC3000 carrying HopZ1a as HopZ1a elicits a strong HR in Arabidopsis that is dependent on an intact acetyltransferase catalytic triad (Lewis et al., 2008). HopZ1a's acetyltransferase activity is necessary for its recognition by the ZED1 pseudokinase and the ZAR1 resistance protein (Lewis et al., 2008, 2010, 2013; Schreiber et al., 2016). We found no differences in HopZ1a ETI in plants grown in Resilience or Sunshine soil, suggesting that the acetyltransferase activity of HopZ1a is not affected (Figure 4a). We directly tested for the ability of silicon to impact HopZ1a expression, by growing *P. syringae* carrying HopZ1a in minimal media containing silicon. We found that effector induction was not affected by the addition of potassium silicate to the media (Figure 4c). These data suggest that silicon is unable to interfere with the activity or expression of *P. syringae* type III effector proteins. Resistance conferred by silicon has been primarily observed toward fungal or oomycete pathogens that form a haustorium inside the plant cell. Fungal effectors are believed to be delivered to the host cell across the fungal–host membrane interface within the plant cell (Yi & Valent, 2013), suggesting that fungal or oomycete effector proteins would not contact silicon in the apoplast. In addition, the broad‐spectrum nature of silicon resistance against bacterial, oomycete, and fungal pathogens argues against a specific effect on diverse effector proteins.

Silicon improves host resistance to many fungal and oomycete diseases, including rice blast caused by *Magnaporthe grisea* (Rodrigues et al., 2004), powdery mildew of wheat, cucumber and rose (Belanger, Benhamou, & Menzies, 2003; Fawe, Abou‐Zaid, Menzies, & Belanger, 1998; Remus‐Borel, Menzies, & Belanger, 2005; Samuels, Glass, Ehret, & Menzies, 1991; Shetty et al., 2011), and some bacterial diseases, including bacterial wilt of tomato caused by *Ralstonia solanacearum* (Chen et al., 2015; Ghareeb et al., 2011) and bacterial speck of tomato caused by *Pseudomonas syringae* (Andrade et al., 2013). Silicon, in the form of uncharged monosilicic acid, is absorbed through the roots, moves through the transpiration stream, and eventually is deposited in the cell walls, intercellular spaces, and cell lumens of leaves and other tissues (Epstein, 1994; Guerriero et al., 2016; Kim, Kim, Park, & Choi, 2002; Samuels et al., 1991). We found that Arabidopsis plants grown in Sunshine or Resilience soil contained ~0.5 mg/g of silicon (Figure 5a), indicating that plants grown in Resilience soil did not take up more silicon than plants grown in Sunshine soil. We observed a similar amount of silicon accumulation as previously reported in nontransgenic Arabidopsis seedlings (0.6–1.5 mg/g of silicon) when they were hydroponically grown in silicon‐supplemented media (Montpetit et al., 2012; Vivancos et al., 2015). Our data indicate that silicon concentrations within the plant are not responsible for the resistance observed in plants grown in Resilience soil. Plants grown on Resilience soil exhibited greater biomass than those grown on Sunshine soil (Figure 5b). As this could be due to nutrient or soil composition, we also analyzed the soil properties and elemental content for both Sunshine and Resilience soils (Tables 1 and 2). Resilience soil contained more bioavailable silicon, as well as more bioavailable potassium and sulfur, and more total calcium, magnesium, and sodium compared to Sunshine soil (Table 2). The pH of Sunshine soil was slightly higher compared to Resilience soil, which can also affect the bioavailability of elements (Barber, 1995). The moisture content of Resilience soil was also slightly higher than that of Sunshine soil. These data suggest that additional differences between Resilience and Sunshine soil help promote resistance to *P. syringae* when Arabidopsis plants are grown in Resilience soil. It is also possible that plants grown in Resilience soil are more resistant because they have more vegetative resources to devote to defenses, similar to the resource reallocation that has been proposed to occur during the growth–defense trade‐off (Huot, Yao, Montgomery, & He, 2014).

Plant immunity can be primed for rapid defenses to a later infection by applying chemicals such as SA (Conrath et al., 2006), azelaic acid (Jung, Tschaplinski, Wang, Glazebrook, & Greenberg, 2009), and pipecolic acid (Bernsdorff et al., 2016). There is a cost to inducing defenses as this redirects resources away from growth, leading to smaller plants and reduced photosynthetic capacity (Bernsdorff et al., 2016; Bowling, Clarke, Liu, Klessig, & Dong, 1997; Mateo et al., 2006; Mauch et al., 2001). As SA is a major contributor to systemic broad‐spectrum resistance in plants, we tested whether mutants impaired in the production of salicylic acid or its signaling displayed greater resistance when grown in Resilience soil versus Sunshine soil. We observed higher levels of bacterial growth in *ics1*,* npr1,* and *nahG* lines compared to Col‐0, consistent with previous reports (Cao et al., 1994; Delaney et al., 1994; Lewis et al., 2010; Nawrath & Metraux, 1999). *ics1*,* npr1,* and *nahG* lines grown in Resilience soil supported less bacterial growth than plants grown in Sunshine soil, and the difference in bacterial growth on plants grown in Sunshine or Resilience soil was similar between Col‐0 and *ics1*,* npr1,* or *nahG* lines (Figure 6). This suggests that enhanced resistance associated with plant growth in Resilience soil is SA‐independent.

Historically, different soil types were known to have disease suppressive properties and to inhibit disease by root‐colonizing pathogens (Chet & Baker, 1981; Lifshitz, Sneh, & Baker, 1984; Martin & Hancock, 1986; Mazzola, 2002; Schroth & Hancock, 1982). Metagenomic analysis of the soil and rhizosphere microbiome suggests that microbial communities may compete with root‐colonizing pathogens to prevent disease, actively inhibit pathogens, and/or promote plant health or immune responses (De Coninck et al., 2015; Hadar & Papadopoulou, 2012; Mendes, Garbeva, & Raaijmakers, 2013). In addition, chemical and physical attributes of the soil can influence the soil microbiome (Hadar & Papadopoulou, 2012). Interestingly, our data show that the plants' soil environment can influence the resistance of plants to foliar pathogens, and the resistance is SA‐independent (Figures 1, 2 and 6). Plant growth‐promoting bacteria in the rhizosphere have previously been shown to trigger ISR in aerial portions of the plant that are inoculated with a different pathogen (Alstrom, 1991; Gang, Kloepper, & Tuzun, 1991; Pieterse et al., 2014; Van Peer, Niemann, & Schippers, 1991). Although ISR is SA‐independent (Pieterse, vanWees, Hoffland, vanPelt, & vanLoon, 1996), NPR1 appears to have a unique role in ISR compared to SAR (Pieterse et al., 2014; Spoel et al., 2003; Stein, Molitor, Kogel, & Waller, 2008). Our data showed that *npr1* plants grown in Resilience soil still exhibited significantly enhanced bacterial resistance compared to plants grown in Sunshine soil (Figure 6). Thus, it is not clear whether Resilience soil may contain plant growth‐promoting bacteria that trigger ISR. We cannot exclude the possibility that silicon or other elements in the soil (Table 2) may affect the microbiome, which may then promote plant resistance. Regardless, our work demonstrates the importance of environmental factors such as soil properties, in contributing to plant resistance to pathogens. In addition, our work highlights the significance of careful documentation of the environmental conditions under which plant–microbe interactions are studied, as soil properties can have substantial effects on resistance to pathogenic bacteria.

## AUTHOR CONTRIBUTIONS

JAH and JDL conceived and designed the experiments. JAH, RT‐R, and JDL performed the experiments. JAH, RT‐R, JCW, and JDL analyzed the data. JAH, JCW, and JDL wrote the manuscript.
