# Supplementary material for: Soil mixture composition alters Arabidopsis susceptibility to Pseudomonas syringae infection
Source: Plant Direct. 2018 Feb 19;2(2):e00044. doi: 10.1002/pld3.44 (PMC6508533; doi:10.1002/pld3.44)
Supplement: Supplementary file 1 [file PLD3-2-e00044-s001.pdf]

Reviewer Comments:
Reviewer #2:
Comments to the Author (Required):
I appreciate the thoughtful and thorough response. The changes made to the manuscript addressed all of my questions / concerns.
